# Parkin acts as a transcription factor modulating presenilin-1 and presenilin-2 promoter transactivations

**DOI:** 10.1186/1750-1326-8-S1-P56

**Published:** 2013-10-04

**Authors:** Eric Duplan, Jean Sévalle, Julien Viotti, Thomas Goiran, Charlotte Bauer, Paul Renbaum, Ephrat Levy-Lahad, Clément A Gautier, Olga Corti, Nathalie Leroudier, Frédéric Checler, Cristine Alves da Costa

**Affiliations:** 1 IPMC-CNRS UMR 7275, Valbonne, France; 2 Medical Genetics Institute, Shaare Zedec Medical Center, Hebrew University Medical School, Jerusalem, Israel; 3 INSERM U679, Hôpital de la Pitié-Salpêtrière, Paris, France

## Background

Parkin is associated to autosomal recessive early-onset Parkinson's disease. Parkin acts as an E3-ubiquitin ligase involved in the proteasome-mediated degradation of various substrates. It has been suggested that pathogenic mutations of parkin, abolishing its ubiquitin-ligase activity, could explain the accumulation of proteins and lead to neuronal death by apoptosis. However, besides this function, additional parkin-dependent cellular pathways exist. We demonstrated that parkin is a direct transcriptional repressor of the tumor suppressor p53 [1]. p53 regulates the expression and functions of presenilin-1 (PS1) and presenilin-2 (PS2), two members of the gamma secretase complex involved in the production of the amyloid β peptide (Aβ) and parkin could control the homeostasis of intracellular Aβ. These findings prompted us to investigate whether parkin could control presenilins and if so, whether it is via a direct transcriptional control of PS promoters or indirectly, via p53.

## Materials and methods

Experiments were conducted on TSM1 neurons, SH-SY5Y human neuroblastoma cells, human embryonic kidney 293 cells, and immortalized mouse embryonic fibroblasts invalidated or not for *parkin*, *presenilin 1* and/or *2*, *p19^arf^* and both *p19^arf^ p53.* We also used primary cultured neurons and brain extract from mouse invalidated or not for *parkin.*

We did Q-PCR, Western-blot, caspases-3 activity measurement and in vitro gamma secretase assays experiments. We document by chromosome immune-precipitation, gel shift, gene reporter and mutagenesis experiments parkin direct interaction with presenilins promoters.

## Results

Parkin controls presenilin 1 and 2 expressions, promoter activity, and mRNA levels *ex vivo* and in mouse brains. This regulation impacts on PS-dependent γ-secretase activity and presenilin-mediated control of cell death. This control is independent of parkin ubiquitin-ligase activity, does not involve p53 and is not affected by PS1 and PS2 functional interplay. Parkin binds to presenilins promoters via a consensus binding sequence that we identify and validate by functional analysis [2].

## Conclusions

This study is a "framework" for the identification of novel transcriptional targets of parkin and for a better comprehension of parkin's functions.
